# Medical Students in the United States during the Session of 1857–58

**Published:** 1858-05

**Authors:** 


### Editors' Table.

MEDICAL STUDENTS IN THE UNITED STATES DURING THE SESSION OF 1857–58.—We publish a list of the students and graduates of the different schools, which, although reliable, must necessarily be imperfect from the fact of our not being in possession of all the catalogues and circulars, anxious as we have been to obtain them:—

| | Students. | Graduates. |
|---|---|---|
| Jefferson Medical College | 501 | 209 |
| University of Pennsylvania | 435 | 145 |
| University of New York | — | 127 |
| University of Nashville | 353 | 109 |
| University of Louisiana | 276 | 68 |
| Georgia Medical College | — | 61 |
| College of Physicians and Surgeons | — | 53 |
| St. Louis Medical College | 125 | 49 |
| Medical College of Ohio | — | 43 |
| Rush Medical College | 100 | 36 |
| Pennsylvania Medical College | 140 | 35 |
| New Orleans School of Medicine | 126 | 33 |
| New York Medical College | — | 33 |
| University of Louisville | — | 31 |
| University Medical College | — | 27 |
| Missouri Medical College | — | 25 |
| Memphis Medical College | — | 19 |
| Philadelphia College of Medicine | 63 | 18 |
| Massachusetts Medical College | — | 16 |
| Transylvania University | — | 12 |
| Oglethorpe Medical College | 37 | 11 |
| Kentucky School of Medicine | — | 11 |
| Starling Medical College | — | 10 |
| University of Buffalo | — | 9 |
| Medical Department of Yale College | — | 6 |
| Medical College of South Carolina | 276 | — |
| Richmond Medical College | 60 | — |
| University of Virginia | 88 | — |

*Students in Philadelphia.*—From the above table it will be seen that there were 1139 students in Philadelphia during the past session. Of these 501 attended the Jefferson Medical College; 435 the University of Pennsylvania; 140 the Pennsylvania College; and 63 the Philadelphia College. The number of graduates in the same schools amounted to 407, viz. at the Jefferson College 209; at the University 145; at the Pennsylvania College 35; and at the Philadelphia College 18.

